# Pharmacological melatonin induces mitochondrial dysfunction and alters extracellular nucleotide metabolism and CD39/CD73 expression in human melanoma cells

**DOI:** 10.1007/s11302-026-10193-z

**Published:** 2026-10-20

**Authors:** Bruna Cristina Ozelame, Daiane Mânica, Gilnei Bruno da Silva, Andreia Machado Cardoso, Margarete Dulce Bagatini, Aline Mânica

**Affiliations:** 1 Post-Graduate Program in Health Sciences, Community University of the Region of Chapecó (UNOCHAPECÓ), Chapecó, SC 89809-900 Brazil; 2 Postgraduate Program in Biochemistry, Federal University of Santa Catarina (UFSC), Florianópolis, Brazil; 3 Multicenter Postgraduate Program in Biochemistry and Molecular Biology, State University of Santa Catarina (UDESC), Lages, Brazil; 4 Post-Graduate Program in Biomedical Sciences, Federal University of Fronteira Sul, Chapecó, Brazil

**Keywords:** Skin cancer, Cytostatic agents, In vitro tests, Nucleotides, Mitochondrial dysfunction, Immune responses, Purinergic agents

## Abstract

Cutaneous melanoma remains an aggressive malignant neoplasm characterized by molecular heterogeneity and therapeutic resistance, highlighting the need to identify alternative pathways involved in tumor progression. Purinergic signaling, particularly the conversion of extracellular ATP to immunosuppressive adenosine mediated by ectonucleotidases CD39 (NTPDase1) and CD73 (ecto-5′-nucleotidase), has emerged as a critical determinant of melanoma biology, but its potential regulation by melatonin remains unexplored.Here, we investigated whether pharmacological melatonin (0.1–5 mM, 24 and 48 h) influences metabolic activity, mitochondrial homeostasis, and extracellular nucleotide metabolism in two human melanoma cell lines with distinct molecular profiles (SK-MEL-28 and A375). Melatonin reduced metabolic activity and induced mitochondrial depolarization in both cell lines, although A375 cells exhibited greater mitochondrial sensitivity than SK-MEL-28 cells. These mitochondrial alterations were accompanied by distinct apoptosis- and inflammation-related transcriptional responses, indicating lineage-dependent cellular response to pharmacological melatonin. In parallel, melatonin differentially altered extracellular nucleotide metabolism and CD39/CD73 gene expression, with distinct responses observed between the two melanoma cell lines. Collectively, these findings indicate that the biological effects of pharmacological melatonin extend beyond mitochondrial dysfunction and involve remodeling of extracellular nucleotide metabolism, providing new evidence for an interaction between melatonergic and purinergic signaling in melanoma.

**Graphical Abstract:**

**Figure:**
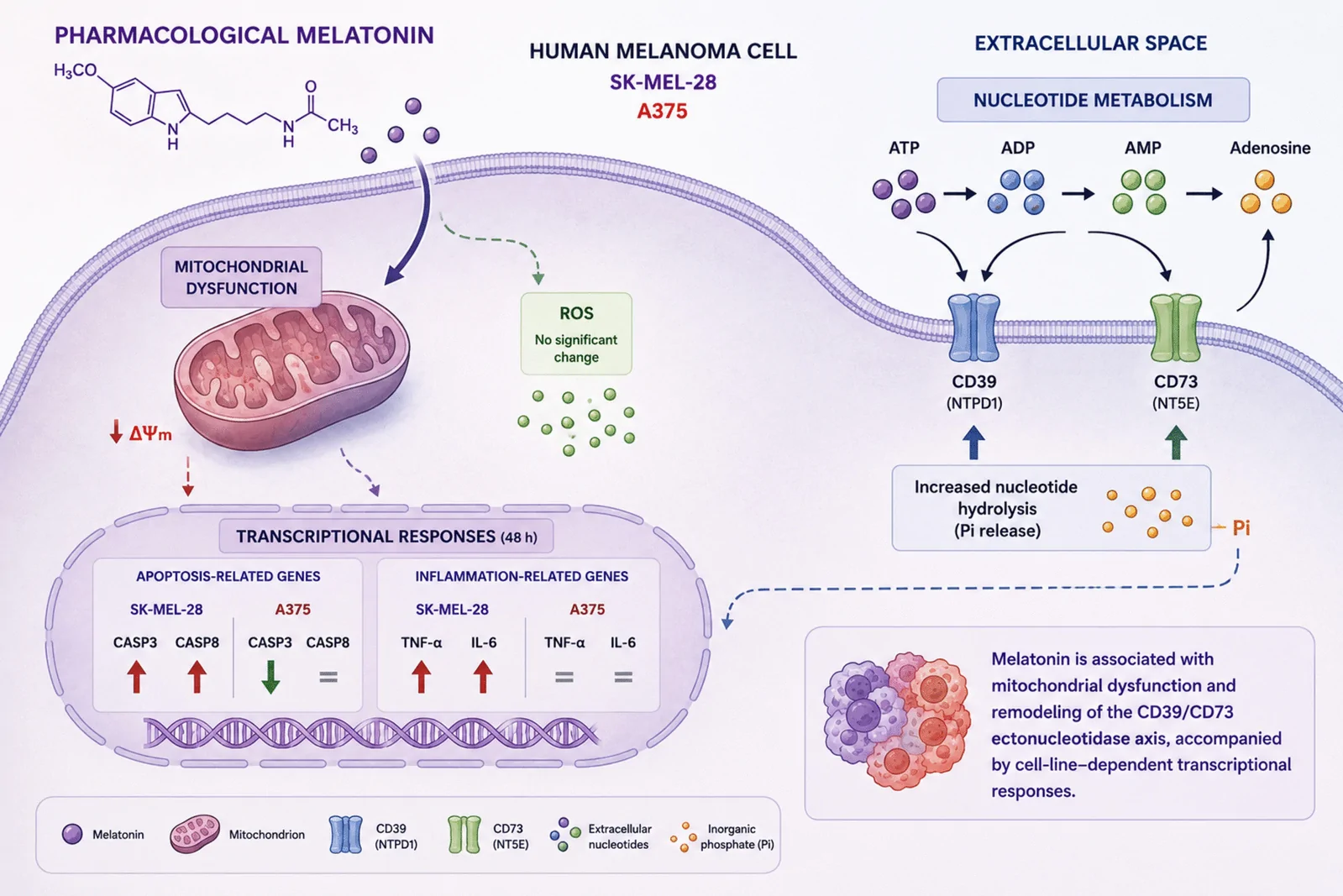

## Introduction

Cutaneous melanoma is one of the most aggressive forms of skin cancer, characterized by high metastatic potential, remarkable molecular heterogeneity, and intrinsic or acquired resistance to current therapeutic strategies, including BRAF-targeted therapy and immune checkpoint inhibitors [1, 2]. Although these approaches have substantially improved patient outcomes, durable clinical responses remain limited for a considerable proportion of patients due to therapeutic resistance and tumor adaptation [3, 4]. Consequently, increasing efforts have focused on identifying alternative molecular pathways involved in melanoma progression that may represent novel therapeutic targets. Among these, purinergic signaling and its close relationship with cellular metabolism and inflammatory processes has emerged as an important regulator of tumor behavior [5, 6].

Melatonin (N-acetyl-5-methoxytryptamine) is an indoleamine synthesized not only by the pineal gland but also by several peripheral tissues and cell types, including the skin, immune cells, gastrointestinal tract, and mitochondria [7]. Beyond its well-established role in regulating circadian rhythms [8, 9], melatonin exerts a wide range of biological activities, including the regulation of mitochondrial homeostasis, cellular bioenergetics, redox balance, inflammatory signaling, and metabolic adaptation [10–14]. In cancer, these pleiotropic properties have been associated with antiproliferative, pro-apoptotic, anti-inflammatory, and immunomodulatory effects. Importantly, pharmacological concentrations of melatonin activate mechanisms that extend beyond classical MT1/MT2 receptor signaling, involving alterations in mitochondrial function and cellular metabolism that ultimately influence tumor cell fate [8, 14].

Mitochondria integrate metabolic and signaling pathways that regulate cellular fate and adaptation [15]. In tumor cells, alterations in mitochondrial homeostasis are frequently accompanied by metabolic remodeling and changes in extracellular communication [16]. Because extracellular nucleotide metabolism is closely linked to cellular metabolic status, metabolic adaptations may be accompanied by alterations in purinergic signaling within the tumor microenvironment [17–19]. However, whether pharmacological melatonin affects these interconnected processes in melanoma cells remains poorly understood.

Extracellular nucleotide metabolism is a fundamental component of purinergic signaling and plays a central role in regulating communication between tumor cells and the surrounding microenvironment. Extracellular ATP acts as a danger-associated molecular signal capable of promoting inflammatory and antitumor responses, whereas its sequential hydrolysis generates ADP, AMP, and ultimately adenosine, a potent immunosuppressive metabolite that promotes tumor progression, angiogenesis, and immune evasion [20–24]. This process is primarily regulated by the ectonucleotidases CD39 and CD73, which control the balance between pro-inflammatory nucleotides and immunosuppressive adenosine [25, 26].

Consequently, alterations in extracellular nucleotide metabolism profoundly influence the metabolic and immunological landscape of the tumor microenvironment and have been associated with tumor progression, immune escape, and resistance to anticancer therapies. In cutaneous melanoma specifically, our group has established a consistent line of investigation into the pharmacological modulation of ectonucleotidase activity and the CD39/CD73 axis, demonstrating that structurally diverse bioactive compounds significantly alter NTPDase and ecto-5′-nucleotidase activities in melanoma cells, with downstream consequences for tumor cell viability, migration, and apoptosis [5, 6, 27–31].

Despite growing evidence supporting the antitumor properties of pharmacological melatonin, whether this indoleamine alters extracellular nucleotide metabolism in melanoma cells remains unknown. Considering the central role of the CD39/CD73 ectonucleotidase axis in regulating extracellular nucleotide metabolism, investigating this pathway may provide mechanistic insights into the biological effects of pharmacological melatonin. Furthermore, understanding whether alterations in extracellular nucleotide metabolism are associated with changes in mitochondrial homeostasis may improve our understanding of the metabolic adaptations induced by melatonin in melanoma cells. Therefore, the present study investigated whether pharmacological melatonin alters extracellular nucleotide metabolism and CD39/CD73 gene expression in human melanoma cells and whether these alterations are associated with mitochondrial homeostasis.

## Materials and methods

### Cell culture

The human cutaneous melanoma cell lines SK-MEL-28 and A375 were obtained from the Rio de Janeiro Cell Bank (BCRJ, Brazil). Cells were maintained in Dulbecco’s Modified Eagle Medium (DMEM) supplemented with 10% fetal bovine serum (FBS) and penicillin/streptomycin (100 U/mL and 100 µg/mL, respectively). SK-MEL-28 cells were cultured in low-glucose DMEM, while A375 cells were maintained in high-glucose DMEM, both containing L-glutamine. Cultures were incubated at 37 °C in a humidified atmosphere with 5% CO₂ and monitored daily for contamination.

### Melatonin treatment

Melatonin (Sigma-Aldrich®, M5250; molecular formula: C13H16N2O2; molecular weight: 232.28 g/mol) was dissolved in 0.5% ethanol and tested at concentrations of 0.1 mM, 1 mM and 5 mM as described in previous studies [32, 33]. For the negative control, culture medium alone was used, while the vehicle control consisted of 0.5% ethanol in culture medium. Melatonin-treated cells were incubated at 37 °C in a humidified atmosphere with controlled CO₂ levels and analyzed after 24 and 48 h of treatment. All experiments were performed in triplicate to ensure data reliability.

### Metabolic activity assay (MTT)

Metabolic activity was assessed using the MTT assay (3-[4,5-dimethylthiazol-2-yl]−2,5-diphenyltetrazolium bromide), following the protocols described in the literature [34, 35]. MTT is a water-soluble tetrazolium salt that is metabolically reduced by viable cells to insoluble purple formazan crystals, which accumulate due to the impermeability of intact cell membranes. Cells were seeded in 96-well plates and exposed to melatonin for 24 and 48 h. After treatment, MTT reagent (5 mg/mL) diluted in phosphate-buffered saline (PBS, pH adjusted) was added, and cells were incubated for 2 h at 37 °C. Following incubation, 200 µL of dimethyl sulfoxide (DMSO) was added to solubilize the formazan crystals. Absorbance was measured at 570 nm using a Varioskan™ LUX microplate reader (Thermo Fisher Scientific™). Results were expressed as a percentage of metabolic activity relative to the control group.

### Mitochondrial membrane potential (ΔΨm)

Mitochondrial membrane potential (ΔΨm) was evaluated using the tetramethylrhodamine ethyl ester (TMRE) dye. Cells were incubated with TMRE (20 nM) for 30 min at 37 °C, protected from light. Fluorescence images were acquired using an inverted fluorescence microscope (Nikon Eclipse TS2-FL) at 20 × magnification [36]. Image analysis was performed using ImageJ software. Fluorescence intensity, proportional to mitochondrial membrane potential, was normalized to the control group and expressed as percentage. TMRE accumulates in the mitochondrial matrix in proportion to the electrochemical gradient of the inner mitochondrial membrane; therefore, loss of signal reflects dissipation of ΔΨm [37]*.*

### Measurement of reactive oxygen species (ROS)

Intracellular ROS levels were determined using a fluorimetric assay based on 2′,7′-dichlorofluorescein diacetate (DCFH-DA) (Sigma-Aldrich), according to the manufacturer's instructions. Fluorescence intensity, proportional to ROS levels, was measured at excitation/emission wavelengths of 490/520 nm using a Varioskan LUX microplate reader (Thermo Fisher Scientific). Results were normalized to the control group and expressed as percentage of ROS.

### ATP, ADP, and AMP hydrolysis assay

The hydrolysis of ATP, ADP, and AMP was evaluated according to a modified protocol [38, 39]. For ATP and ADP hydrolysis assays, cells were incubated with a buffer containing NaCl, KCl, glucose, Tris–HCl (pH 7.4), CaCl₂, and distilled water. For AMP hydrolysis assays, CaCl₂ was replaced with MgCl₂. Following a 10-min pre-incubation at 37 °C, the appropriate nucleotide (ATP, ADP, or AMP) was added at a final concentration of 1 mM, and cells were incubated for an additional 70 min. Parallel control wells containing cells incubated in reaction buffer without nucleotide substrate were processed under identical conditions; the inorganic phosphate released in these substrate-free wells was subtracted from all experimental measurements to correct for basal, non-substrate-dependent phosphate release.

The reaction was stopped by adding 15% trichloroacetic acid (TCA), and the release of inorganic phosphate (Pi) was quantified using a malachite green colorimetric assay. Absorbance was measured at 630 nm using a Varioskan™ LUX microplate reader (Thermo Fisher Scientific™). All assays were performed in triplicate, and results were expressed as nmol Pi/min/mg protein.

### Gene expression analysis

Gene expression was assessed by quantitative reverse transcription PCR (qRT-PCR) [40]. Total RNA was extracted using TRIzol® reagent (Invitrogen), and concentration and purity were determined by spectrophotometry (Varioskan™ LUX, Thermo Fisher Scientific™) at 260/280 nm. RNA samples were treated with DNase, and cDNA synthesis was performed using the MultiScript™ cDNA Synthesis Kit (Bio-Rad®), according to the manufacturer's instructions. qPCR reactions were carried out using 3 µL of cDNA and PowerUp™ SYBR Green PCR Master Mix (Thermo Scientific®) in a final volume of 10 µL, under standard thermal cycling conditions. Melt curve analysis was performed to confirm amplification specificity. All reactions were run in duplicate. GAPDH was used as the reference gene for normalization [27–29].

We used the following sequences of oligos (5′–3′): GAPDH (f): CTCCTCACAGTTGCCATGTA; GAPDH (r): GTTGAGCACAGGGTACTTTATTG; CASP3 (f): TTTGAGCCTGAGCAGAGACATG; CASP3 (r):TACCAGTGCGTATGGAGAAATGG; CASP8 (f): AGGAGCTGCTCTTCCGAATT; CASP8 (r): CCCTGCCTGGTGTCTGAAGT; TNF-ⲁ (f): CAGGCAGTCAGATCATCTTC; TNF-ⲁ (r): GCTTGAGGGTTTGCTACAAC; IL-6 (f): TCATCCCATAGCCCAGAGCA; IL-6 (r): CTGGCATTTGTGGTTGGGTC; CD39 (f): GCCCTGGTCTTCAGTGTATTAG; CD39 (r): CTGGCATAACCTACCTACTCTTTC; CD73 (f): GTGCCTTTGATGAGTCAGGTAG; CD73 (r): TTCCTTCTCTCGTGTCCTTTG.

### Statistical analysis

Statistical analyses were performed using GraphPad Prism 10 software. Samples were analyzed using the one-way analysis of variance (ANOVA) followed by the Student–Newman–Keuls post hoc test for multiple comparisons. When appropriate, comparisons between two groups were performed using the Student’s t-test. Results were considered statistically significant when *p* < 0.05. Significance levels were indicated as: *p < 0.05; **p < 0.01; ***p < 0.001; ****p < 0.0001.

## Results

**Fig. 1:**
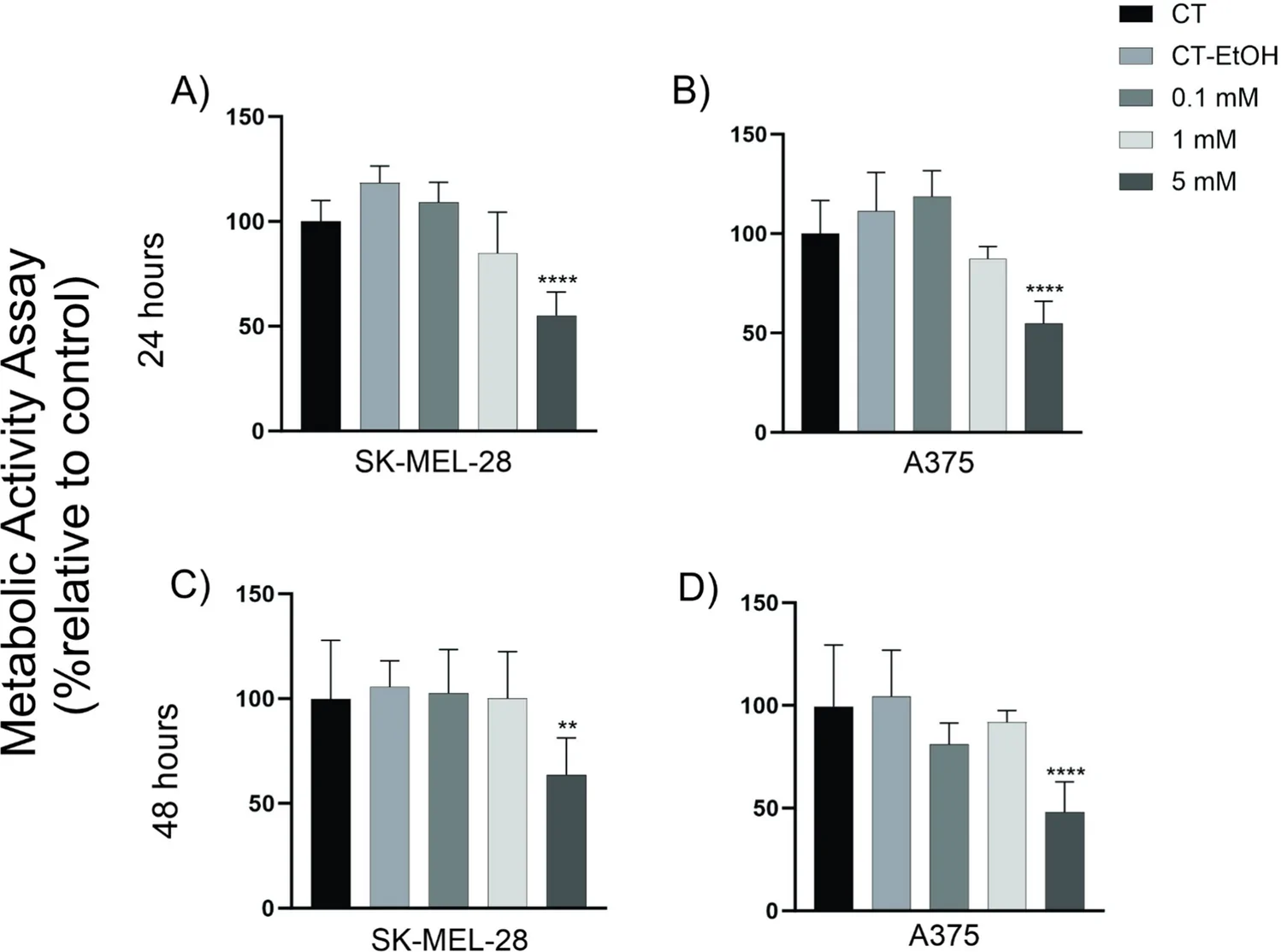
Effect of pharmacological melatonin on the metabolic activity of SK-MEL-28 and A375 melanoma cells after 24 h (**A**) and (**B**) and 48 h (**C**) and (**D**). Metabolic activity was determined using the MTT assay and expressed as a percentage relative to untreated control cells (CT). An ethanol vehicle control (CT-EtOH), corresponding to the solvent used to dissolve melatonin, was included. Panels A and C represent SK-MEL-28 cells, whereas panels B and D correspond to A375 cells. Data are presented as mean ± SD. Statistical significance relative to the untreated control is indicated (**p < 0.01; ****p < 0.0001)

Exposure to pharmacological melatonin reduced the metabolic activity of both melanoma cell lines in a concentration-dependent manner (Fig. 1). No significant changes were observed following treatment with 0.1 or 1 mM melatonin after either 24 or 48 h. In contrast, treatment with 5 mM melatonin significantly reduced metabolic activity in both SK-MEL-28 and A375 cells.

In SK-MEL-28 cells, exposure to 5 mM melatonin significantly decreased metabolic activity after both 24 h (Fig. 1A, *p* < 0.0001) and 48 h (Fig. 1C, *p* < 0.01) compared with untreated controls. Similarly, A375 cells exhibited a marked reduction after 24 h and 48 h of treatment (Fig. 1B and D, *p* < 0.0001). Under these conditions, metabolic activity was reduced by approximately 50% in both melanoma cell lines relative to the control group. No significant differences were observed between the ethanol vehicle control and untreated cells, indicating that the solvent did not influence the observed effects.

**Fig. 2:**
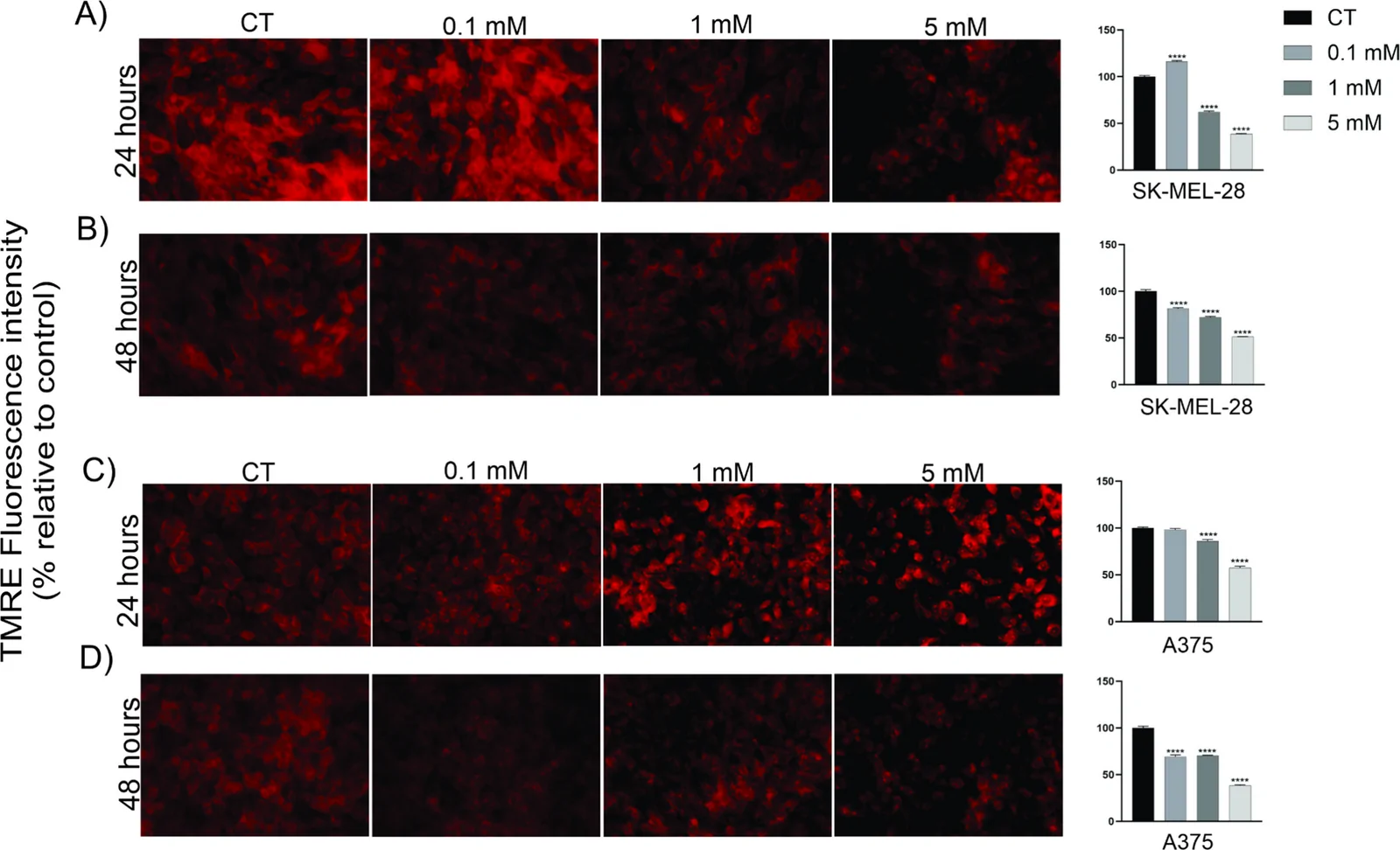
Effect of pharmacological doses of melatonin on mitochondrial membrane potential (ΔΨm) in SK-MEL-28 and A375 melanoma cells after 24 h and 48 h of treatment. ΔΨm was assessed via TMRE fluorescence and expressed as a percentage relative to untreated control cells (CT). Panels (**A**) and (**B**) correspond to SK-MEL-28 cells at 24 h and 48 h, respectively, while panels (**C**) and (**D**) correspond to A375 cells at 24 h and 48 h, respectively. Representative fluorescence images are shown alongside the respective quantitative analyses. Data are presented as mean ± SD. Statistical significance relative to the untreated control is indicated by asterisks (*p < 0.05; **p < 0.01; ***p < 0.001; ****p < 0.0001)

Given the close relationship between cellular metabolic activity and mitochondrial function, mitochondrial membrane potential (ΔΨm) was subsequently evaluated to determine whether melatonin treatment was associated with alterations in mitochondrial homeostasis (Fig. 2).

Melatonin induced concentration- and time-dependent changes in ΔΨm in both melanoma cell lines. In SK-MEL-28 cells, treatment with 0.1 mM melatonin significantly increased TMRE fluorescence after 24 h compared with the untreated control (*p* < 0.0001). In contrast, exposure to 1 mM and 5 mM significantly reduced TMRE fluorescence at both 24 h and 48 h (*p* < 0.0001). After 48 h, even the lowest concentration significantly decreased ΔΨm relative to the control.

In A375 cells, no significant change in TMRE fluorescence was observed following treatment with 0.1 mM melatonin after 24 h. However, treatment with 1 mM and 5 mM significantly reduced ΔΨm at both time points (*p* < 0.0001). After 48 h, all melatonin concentrations significantly decreased TMRE fluorescence compared with untreated cells. Overall, these findings demonstrate that pharmacological melatonin is associated with loss of mitochondrial membrane potential in both melanoma cell lines, particularly following prolonged exposure and at higher concentrations.

**Fig. 3:**
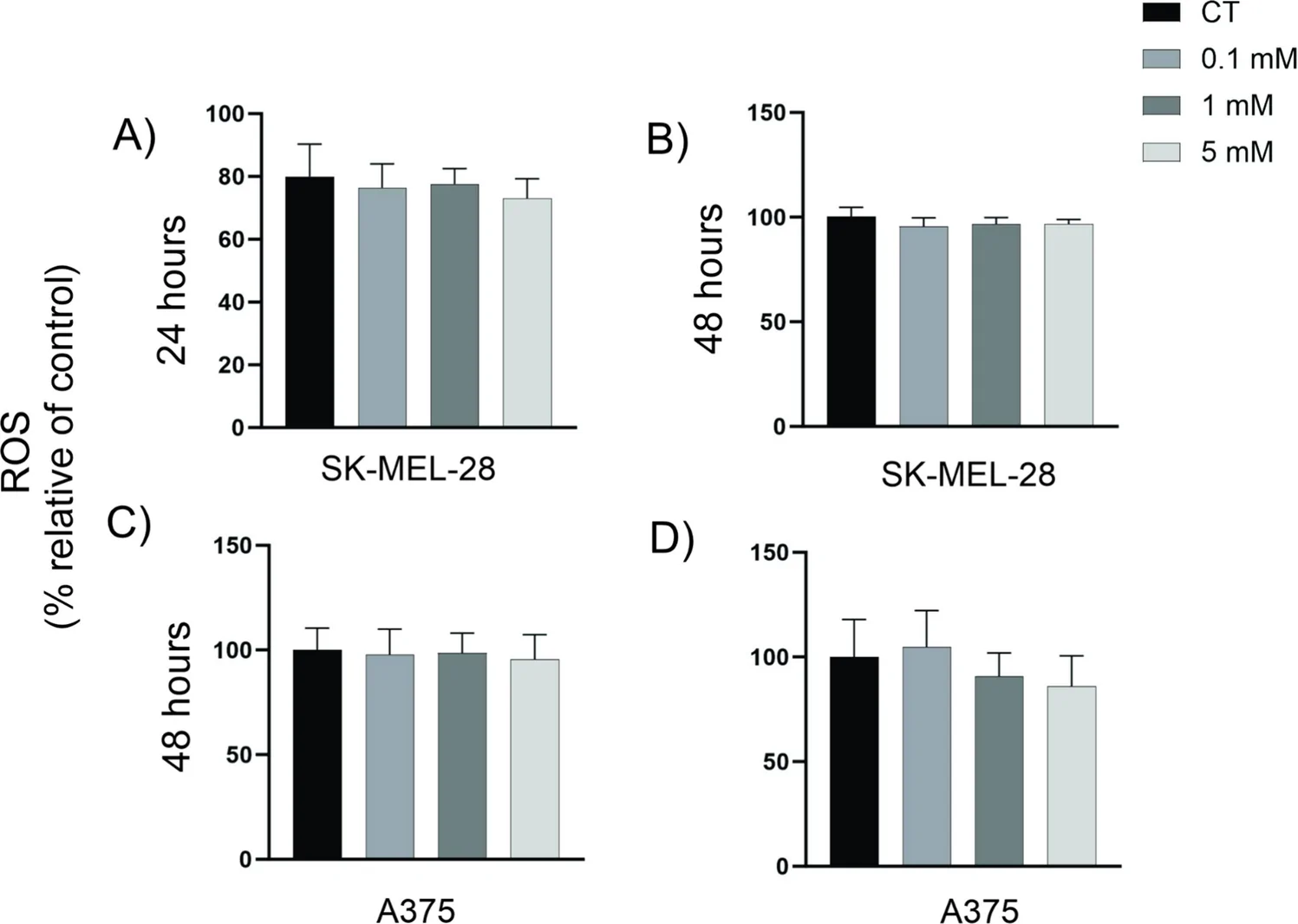
Effect of pharmacological doses of melatonin on intracellular levels of reactive oxygen species (ROS) in SK-MEL-28 and A375 melanoma cells after 24 h (**A**) and (**C**) and 48 h (**B**) and (**D**) of treatment. Intracellular ROS levels were determined using the DCFH-DA fluorescence assay and expressed as a percentage relative to untreated control cells (CT). Panels A and B represent SK-MEL-28 cells, while panels C and D correspond to A375 cells. Data are presented as mean ± SD. No statistically significant differences were observed between the experimental groups

To determine whether the observed loss of mitochondrial membrane potential was accompanied by alterations in intracellular redox status, ROS production was subsequently evaluated (Fig. 3).

Intracellular ROS levels were measured to determine whether the reduction in mitochondrial membrane potential was accompanied by oxidative imbalance (Fig. 3). Melatonin treatment did not significantly alter ROS production in either SK-MEL-28 or A375 cells at any concentration or exposure time evaluated. These findings indicate that the mitochondrial alterations induced by pharmacological melatonin were not accompanied by detectable accumulation of intracellular ROS under the experimental conditions employed.

**Fig. 4:**
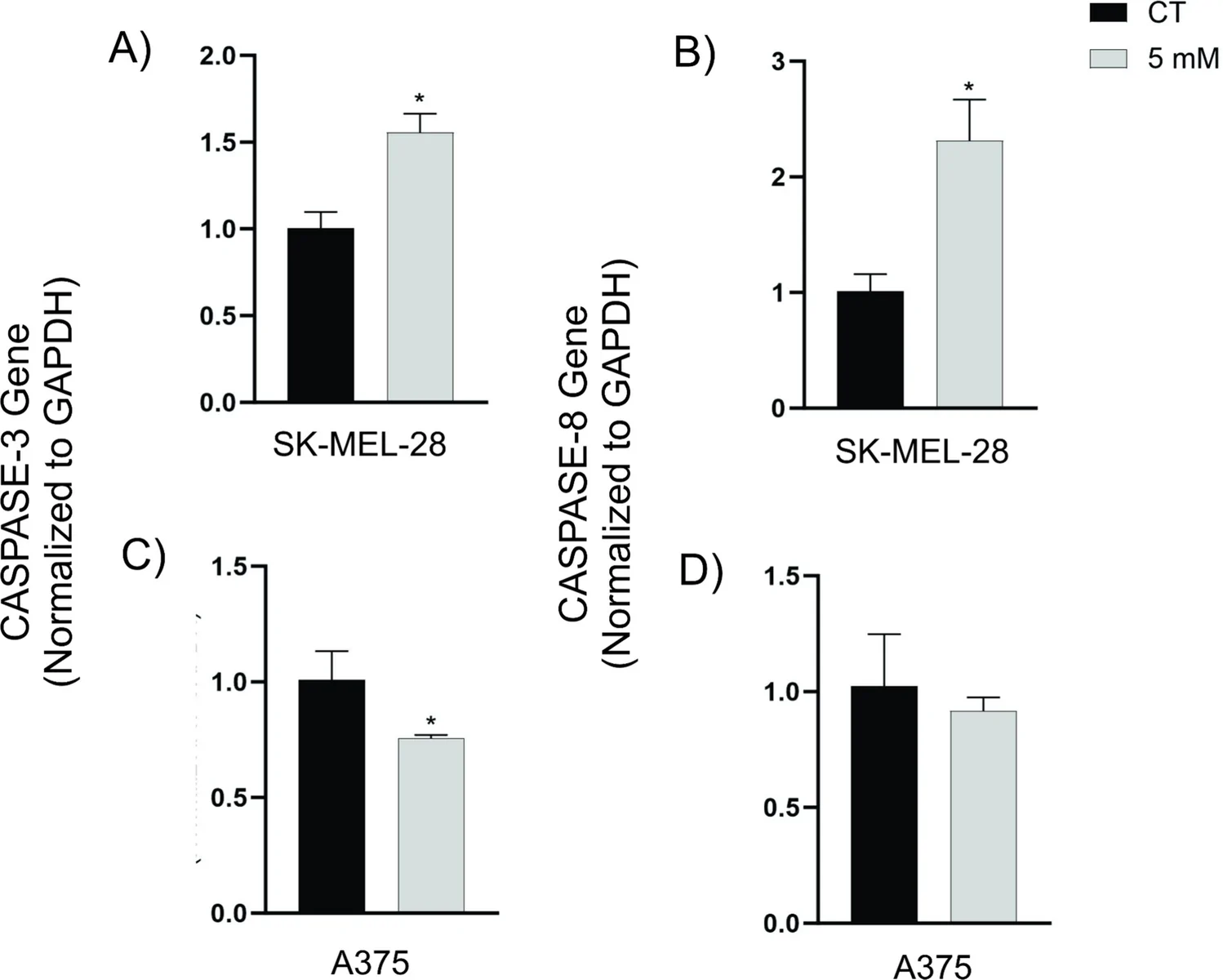
Effect of pharmacological melatonin on the expression of apoptosis-related genes in melanoma cells. CASP3 (**A**) and (**C**) and CASP8 (**B**) and (**D**) mRNA expression was determined by quantitative real-time PCR after 48 h of treatment with 5 mM melatonin. Expression levels were calculated using the ΔΔCt method and normalized to GAPDH. Panels A and B correspond to SK-MEL-28 cells, whereas panels C and D correspond to A375 cells. Data are presented as mean ± SD. *p* < 0.05 versus untreated control (CT)

**Fig. 5:**
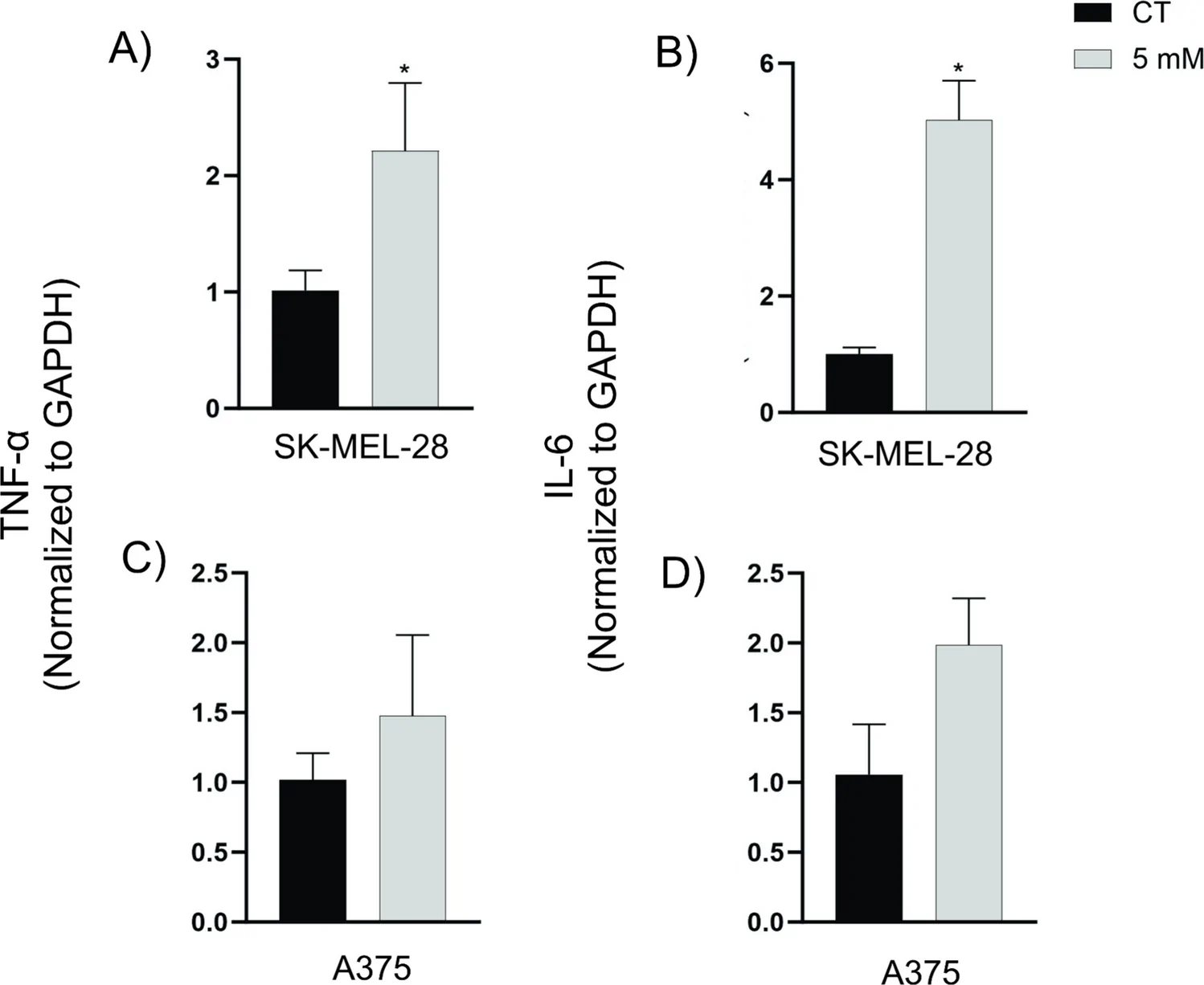
Effect of pharmacological melatonin on the expression of inflammation-related genes in melanoma cells. TNF (**A**) and (**C**) and IL6 (**B**) and (**D**) mRNA expression was determined by quantitative real-time PCR after 48 h of treatment with 5 mM melatonin. Expression levels were calculated using the ΔΔCt method and normalized to GAPDH. Panels A and B correspond to SK-MEL-28 cells, whereas panels C and D correspond to A375 cells. Data are presented as mean ± SD. *p* < 0.05 versus untreated control (CT)

To further characterize the cellular response associated with mitochondrial dysfunction, the expression of apoptosis- and inflammation-related genes was evaluated after 48 h of treatment with pharmacological melatonin (Figs. 4 and 5).

Melatonin treatment differentially affected the expression of apoptosis-related genes in the two melanoma cell lines (Fig. 4). In SK-MEL-28 cells, treatment with 5 mM melatonin significantly increased the expression of both CASP3 and CASP8 compared with untreated controls (Figs. 4A and B; *p* < 0.05). In contrast, A375 cells exhibited a significant decrease in CASP3 expression following melatonin exposure (Fig. 4C; *p* < 0.05), whereas CASP8 expression remained unchanged (Fig. 4D).

The transcriptional response was further investigated by evaluating the expression of inflammation-related genes (Fig. 5). In SK-MEL-28 cells, pharmacological melatonin significantly increased the expression of both TNF and IL6 compared with untreated controls (Figs. 5A and B; *p* < 0.05). In contrast, no significant changes in TNF or IL6 expression were observed in A375 cells following treatment (Figs. 5C and D).

**Fig. 6:**
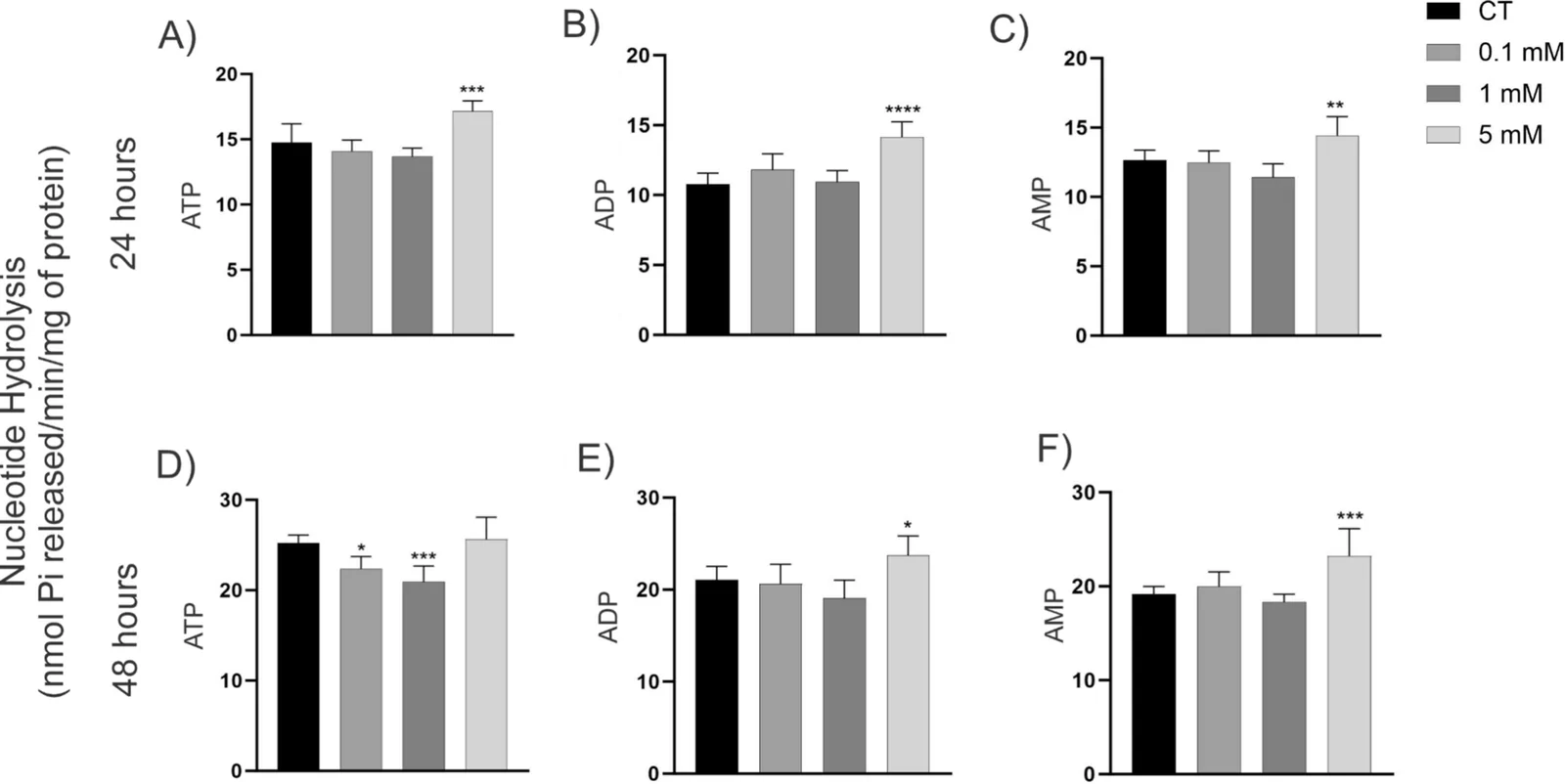
Effect of pharmacological melatonin on extracellular inorganic phosphate (Pi) release from ATP, ADP, and AMP in SK-MEL-28 melanoma cells. Cells were incubated with 1.0 mM ATP (**A**) and (**D**), ADP (**B**) and (**E**), or AMP (**C**) and (**F**) after 24 h (**A**–**C**) or 48 h (**D**–**F**) of melatonin treatment. Pi release was quantified using the malachite green assay and expressed as nmol Pi/min/mg protein. Data are presented as mean ± SD. Statistical significance relative to untreated controls is indicated by asterisks (*p < 0.05; **p < 0.01; ***p < 0.001; ****p < 0.0001)

**Fig. 7:**
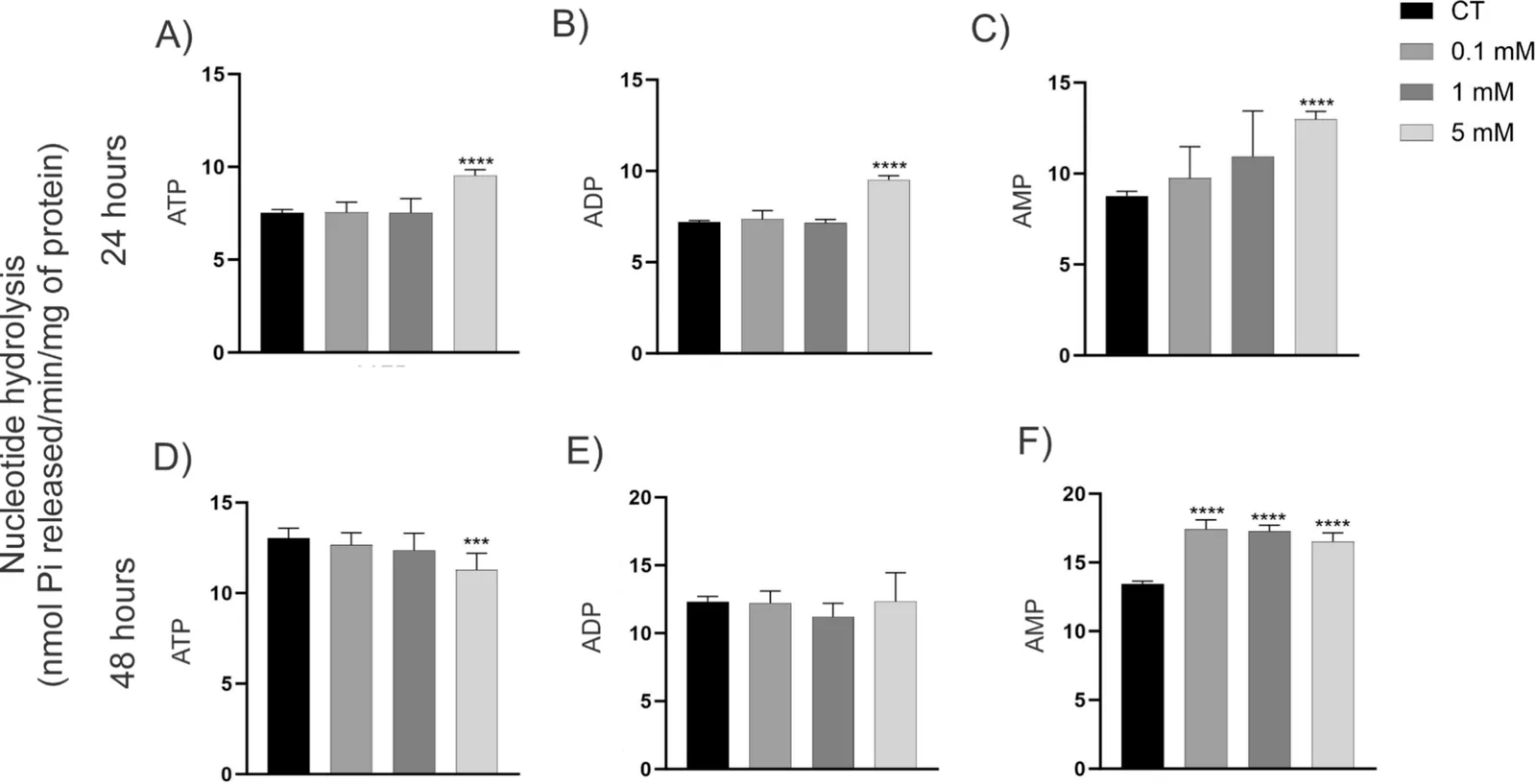
Effect of pharmacological melatonin on extracellular inorganic phosphate (Pi) release from ATP, ADP, and AMP in A375 melanoma cells. Cells were incubated with 1.0 mM ATP (**A**) and (**D**), ADP (**B**) and (**E**), or AMP (**C**) and (**F**) after 24 h (**A**–**C**) or 48 h (**D**–**F**) of melatonin treatment. Pi release was quantified using the malachite green assay and expressed as nmol Pi/min/mg protein. Data are presented as mean ± SD. Statistical significance relative to untreated controls is indicated by asterisks (***p < 0.001; ****p < 0.0001)

Given that inflammatory signaling and cellular metabolic adaptation are closely associated with extracellular nucleotide metabolism, subsequent experiments investigated whether these transcriptional changes were accompanied by alterations in extracellular nucleotide metabolism. Thus, the hydrolysis of ATP, ADP, and AMP was evaluated in both melanoma cell lines after 24 and 48 h of treatment (Figs. 6 and 7).

In SK-MEL-28 cells, pharmacological melatonin differentially affected extracellular nucleotide metabolism depending on treatment duration (Fig. 6). After 24 h, treatment with 5 mM melatonin significantly increased the hydrolysis of ATP, ADP and AMP compared with untreated controls (Figs. 6A–C), whereas no significant changes were observed at lower concentrations. After 48 h, ATP hydrolysis was significantly reduced following treatment with 0.1 and 1 mM melatonin, while no significant difference was observed at 5 mM (Fig. 6D). In contrast, ADP and AMP hydrolysis remained significantly increased following treatment with 5 mM melatonin (Figs. 6E and F).

A375 cells also exhibited alterations in extracellular nucleotide metabolism following melatonin treatment (Fig. 7). After 24 h, exposure to 5 mM melatonin significantly increased the hydrolysis of ATP, ADP, and AMP compared to untreated controls. However, after 48 h of treatment, ATP hydrolysis was significantly reduced at the 5 mM concentration, whereas no significant changes were observed in ADP hydrolysis. Conversely, AMP hydrolysis increased significantly at all melatonin concentrations.

Because extracellular nucleotide metabolism is primarily regulated by the coordinated activity of CD39 and CD73, the expression of these ectonucleotidases was subsequently evaluated to determine whether the functional alterations observed in nucleotide hydrolysis were accompanied by changes in CD39/CD73 gene expression.

**Fig. 8:**
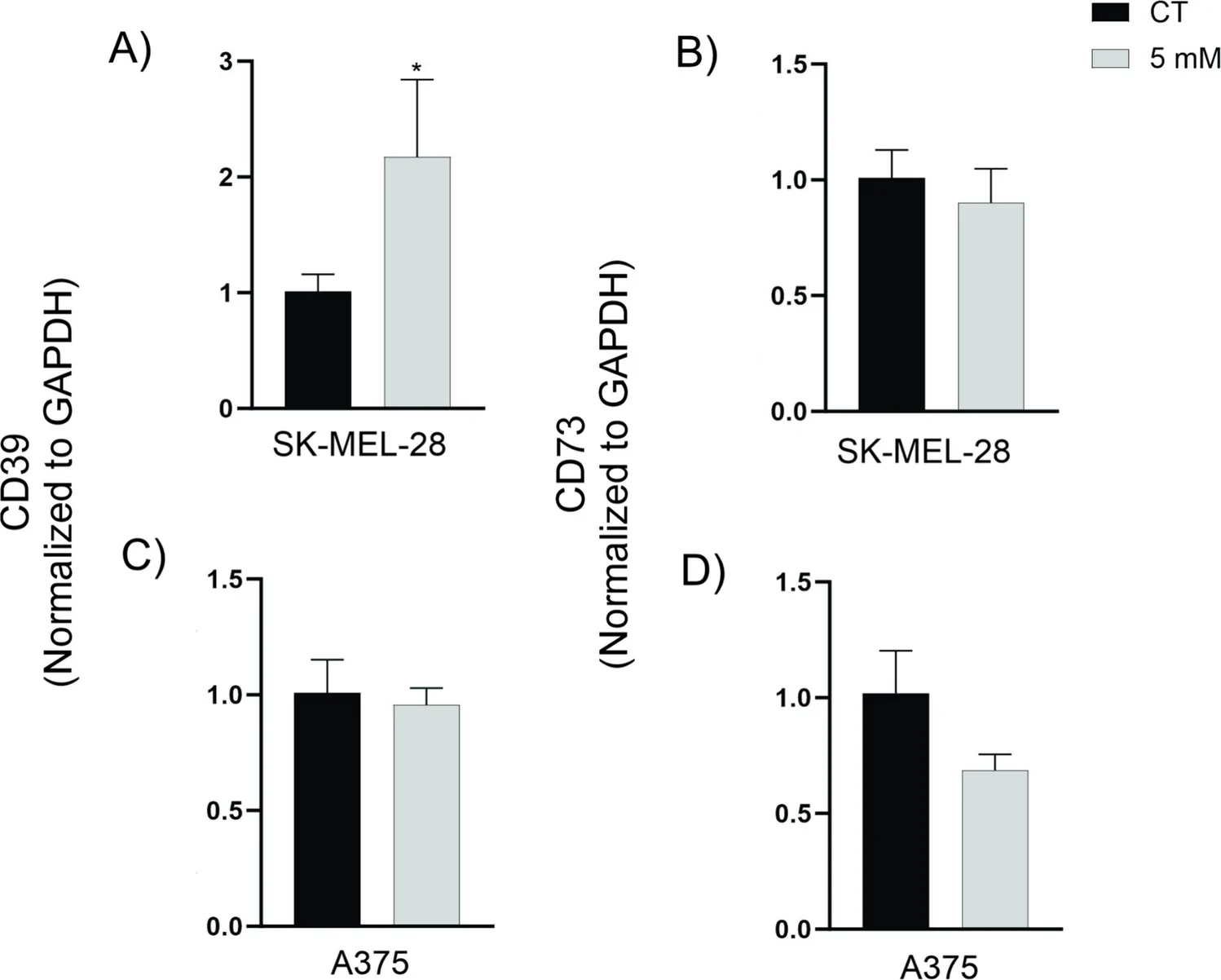
Effect of pharmacological melatonin on CD39 and CD73 gene expression in melanoma cells. CD39 (**A**) and (**C**) and CD73 (**B**) and (**D**) mRNA expression was determined by quantitative real-time PCR after 48 h of treatment with 5 mM melatonin. Gene expression levels were calculated using the comparative ΔΔCt method and normalized to GAPDH. Panels A and B correspond to SK-MEL-28 cells, whereas panels C and D correspond to A375 cells. Data are presented as mean ± SD. *p* < 0.05 versus untreated control (CT)

The expression of CD39 and CD73 was evaluated to determine whether the alterations in extracellular nucleotide metabolism were accompanied by changes in CD39/CD73 gene expression (Fig. 8). In SK-MEL-28 cells, pharmacological melatonin significantly increased CD39 gene expression following 48 h of treatment with 5 mM melatonin compared with untreated controls (Fig. 8A; *p* < 0.05), whereas CD73 expression remained unchanged (Fig. 8B). In contrast, no significant differences in the expression of either CD39 or CD73 were observed in A375 cells after melatonin treatment (Figs. 8C and D).

Together, these findings indicate that the functional alterations in extracellular nucleotide metabolism induced by pharmacological melatonin are accompanied by selective changes in CD39/CD73 gene expression, particularly in SK-MEL-28 cells.

## Discussion

In the present study, pharmacological melatonin reduced the metabolic activity of melanoma cells and was associated with mitochondrial depolarization, cell-line-specific transcriptional responses, and alterations in extracellular nucleotide metabolism. Importantly, these effects were accompanied by differential regulation of CD39/CD73 gene expression, providing evidence that extracellular purine metabolism may be involved in the cellular response of melanoma cells to pharmacological melatonin. Rather than inducing a uniform response across melanoma models, melatonin produced a convergent metabolic and mitochondrial phenotype accompanied by divergent downstream transcriptional and ectonucleotidase-expression responses in SK-MEL-28 and A375 cells. These findings extend previous evidence on the antitumor effects of melatonin and suggest that remodeling of extracellular nucleotide metabolism may represent an additional component of its activity in melanoma [11–14, 20–24].

Although both melanocytes and melanoma cells are capable of synthesizing melatonin [8, 9, 41, 42], most experimental evidence supporting its antitumor effects in melanoma and other cancer models has been generated using pharmacological concentrations [10–14, 43, 44]. Accordingly, the concentrations investigated in the present study were selected to examine pharmacological effects and are within the range previously evaluated in melanoma and other tumor models [24, 33, 45–50]. At pharmacological concentrations, melatonin can exert biological effects that extend beyond its established receptor-mediated actions, including alterations in mitochondrial function, cellular metabolism, and stress-responsive pathways [10–14]. Therefore, the present experimental model was designed to investigate cellular responses associated with pharmacological melatonin rather than to reproduce physiological circulating concentrations.

Pharmacological melatonin significantly reduced metabolic activity in both melanoma cell lines (Fig. 1), consistent with previous reports describing antitumor effects of melatonin in gastric, lung, prostate, ovarian, osteosarcoma, epidermoid carcinoma, and hematological cancer models at pharmacological concentrations ranging from 1 to 10 mM [24, 33, 45–50]. The magnitude of the reduction was already evident after 24 h and remained comparable after 48 h, suggesting that melatonin induces a relatively rapid alteration in cellular metabolic activity that persists during the treatment period. Because the MTT assay reflects cellular metabolic activity, with a substantial contribution from mitochondrial reducing capacity, rather than directly measuring cell number or cell death, these findings should be interpreted as evidence of metabolic impairment rather than definitive evidence of reduced viability or apoptosis. Importantly, the ethanol vehicle did not significantly affect metabolic activity in either cell line, supporting the interpretation that the observed changes were attributable to melatonin treatment rather than solvent toxicity.

The reduction in metabolic activity was accompanied by alterations in mitochondrial membrane potential (Fig. 2). Melatonin induced a concentration-dependent reduction in ΔΨm, although the response differed between the two melanoma cell lines. SK-MEL-28 cells exhibited significant mitochondrial depolarization at 1 and 5 mM, whereas A375 cells responded at all concentrations tested. Because the mitochondrial membrane potential is required for oxidative phosphorylation and maintenance of mitochondrial bioenergetic function, its disruption is widely recognized as an indicator of altered mitochondrial homeostasis [12–14]. Thus, the present findings indicate that pharmacological melatonin affects mitochondrial function under the experimental conditions used and provide a cellular context for the reduction in metabolic activity observed in both melanoma models.

An interesting feature of the mitochondrial response was observed at the lowest concentration tested in SK-MEL-28 cells. At 0.1 mM, melatonin transiently increased TMRE fluorescence after 24 h, whereas a significant reduction in ΔΨm was observed after 48 h. Transient mitochondrial hyperpolarization can occur as part of an adaptive response to cellular perturbation and may precede more pronounced mitochondrial dysfunction. In the present study, however, the temporal sequence of these events was not investigated in sufficient detail to establish causality. Therefore, the transient increase in TMRE fluorescence is best interpreted as evidence of a concentration- and time-dependent mitochondrial response to melatonin rather than as definitive evidence of a compensatory mechanism.

Interestingly, mitochondrial depolarization occurred without detectable increases in intracellular ROS levels (Fig. 3). Although increased ROS generation has been proposed as one mechanism contributing to the antitumor effects of pharmacological melatonin in several experimental cancer models [13], the present findings indicate that mitochondrial dysfunction can occur in melanoma cells in the absence of measurable intracellular ROS accumulation under the conditions tested. This discrepancy may reflect differences in cell type, melatonin concentration, treatment duration, or the contribution of ROS-independent mechanisms to mitochondrial responses.

Because mitochondrial dysfunction may be associated with changes in stress-responsive transcriptional programs independently of measurable ROS accumulation [51], we next examined genes involved in apoptosis-related and inflammatory signaling. In SK-MEL-28 cells, melatonin increased the expression of CASP8 and CASP3 (Fig. 4). Caspase-8 functions primarily as an initiator of death receptor-associated apoptotic signaling, whereas caspase-3 is a major executioner caspase involved in the proteolytic phase of apoptosis [52]. The concurrent increase in CASP8 and CASP3 expression, together with reduced metabolic activity and mitochondrial depolarization, supports the involvement of apoptosis-associated transcriptional responses in SK-MEL-28 cells following melatonin exposure. Importantly, these findings do not establish caspase activation or apoptotic cell death, as changes in mRNA abundance alone are insufficient to demonstrate these processes.

The inflammatory response also differed between the two melanoma cell lines. In SK-MEL-28 cells, 5 mM melatonin significantly increased TNF-α and IL-6 expression (Fig. 5), whereas these transcripts were not significantly altered in A375 cells. TNF-α is a pleiotropic inflammatory mediator that can also participate in death receptor-associated signaling, while IL-6 is involved in inflammatory responses and cellular adaptation to stress [20, 53, 54]. The concurrent increase in TNF-α, IL-6, CASP8, and CASP3 expression in SK-MEL-28 cells therefore supports a cell-line-specific transcriptional response involving inflammatory and apoptosis-associated pathways following pharmacological melatonin exposure.

In contrast, A375 cells displayed reduced CASP3 expression without significant changes in CASP8, TNF-α, or IL-6 expression (Figs. 4 and 5). This transcriptional response occurred despite A375 cells exhibiting pronounced mitochondrial depolarization, indicating that comparable mitochondrial alterations can be accompanied by distinct downstream transcriptional responses. The decrease in CASP3 expression may reflect differential regulation of apoptotic machinery in A375 cells, but its functional significance cannot be established from transcriptional data alone. Thus, the present findings do not allow discrimination between altered apoptotic signaling and the potential involvement of other cellular stress or cell-death pathways. Whether the mitochondrial response observed in A375 cells is associated with other cell-death pathways or adaptive responses remains to be determined.

The divergent transcriptional responses of SK-MEL-28 and A375 cells are consistent with the marked biological heterogeneity of melanoma. Although both cell lines displayed reduced metabolic activity and mitochondrial depolarization following melatonin exposure, their responses differed substantially at the level of apoptosis-associated and inflammatory gene expression. Previous characterization of melanoma models has demonstrated molecular differences between these cell lines, including differences in phenotypic state and expression of genes associated with proliferation and invasion [55–58]. Such intrinsic differences may contribute to distinct downstream responses to mitochondrial perturbation and may contribute to the heterogeneous effects of pharmacological melatonin observed in the present study.

Given these mitochondrial and transcriptional responses, we next examined whether pharmacological melatonin also affects extracellular nucleotide metabolism. In melanoma models, our group has previously investigated the pharmacological modulation of ectonucleotidase activity and the CD39/CD73 axis, demonstrating that structurally diverse bioactive compounds can significantly alter NTPDase and ecto-5′-nucleotidase activities in melanoma cells, with associated changes in tumor cell viability, migration, and apoptosis [5, 6, 27–31].

Extracellular nucleotides are dynamically regulated under conditions of cellular perturbation and can participate in signaling within the tumor microenvironment [20–24]. The CD39/CD73 ectonucleotidase axis is a key pathway for the sequential hydrolysis of extracellular ATP and ADP to AMP by CD39, followed by the conversion of AMP to adenosine by CD73. Extracellular ATP can function as a danger-associated molecular pattern (DAMP), promoting inflammatory and immune-related signaling, whereas its metabolism toward adenosine can favor immunosuppressive processes within the tumor microenvironment. Adenosine signaling has been associated with tumor progression, angiogenesis, and immune evasion [59–62]. Thus, changes in CD39/CD73 activity or expression may influence the balance between extracellular nucleotide-mediated signaling and adenosine-associated responses.

The present findings indicate that pharmacological melatonin alters extracellular nucleotide metabolism in a cell-line-dependent manner rather than uniformly increasing or suppressing ectonucleotidase activity. In SK-MEL-28 cells, melatonin increased the hydrolysis of ATP, ADP, and AMP (Fig. 6) and selectively increased CD39 gene expression, whereas CD73 expression remained unchanged (Fig. 8). Because the hydrolysis assay does not distinguish among ectonucleotidases, this increase in nucleotide-hydrolyzing activity cannot be specifically attributed to CD39; nonetheless, the concurrent rise in CD39 transcription is consistent with a contribution from this enzyme to the observed response.

Importantly, increased nucleotide hydrolysis does not by itself establish changes in extracellular nucleotide availability or purinergic receptor activation, as extracellular nucleotide concentrations and downstream receptor signaling were not directly assessed. Likewise, because extracellular adenosine concentrations and CD73 enzymatic activity were not measured, these findings cannot establish whether melatonin increases net adenosine production. Rather, they indicate that pharmacological melatonin modifies extracellular nucleotide-processing activity in SK-MEL-28 cells, with potential implications for the balance between ATP- and adenosine-mediated signaling.

One potential mechanism linking altered extracellular nucleotide metabolism to the cellular responses observed here involves purinergic receptors, particularly P2X7. Extracellular ATP can activate P2X7, a ligand-gated ion channel expressed in human melanoma cells and associated with calcium-dependent cellular responses, mitochondrial alterations, and inflammatory signaling [63–66]. P2X7 activation has also been linked to inflammasome-associated inflammatory responses in tumor and melanocytic cells [67, 68]. Thus, the cell-line-dependent changes in extracellular nucleotide processing observed in the present study raise the possibility that altered purinergic signaling contributes to the mitochondrial and inflammatory responses to pharmacological melatonin.

Importantly, increased extracellular nucleotide hydrolysis does not by itself establish whether purinergic receptor stimulation is increased or reduced, as receptor signaling depends on the balance between nucleotide availability, ectonucleotidase activity, receptor expression, and local extracellular concentrations. This consideration is particularly relevant for P2X7, which generally requires relatively high extracellular ATP concentrations for robust activation [67]. Therefore, P2X7 involvement remains a mechanistic hypothesis that warrants direct investigation rather than an established mechanism supported by the present data.

The response of A375 cells further emphasizes the cell-line specificity of this process. Despite alterations in nucleotide hydrolysis, no significant changes in CD39 or CD73 gene expression were detected in A375 cells (Figs. 7 and 8). Thus, functional changes in extracellular nucleotide metabolism can occur without detectable transcriptional changes in the two principal ectonucleotidases examined here. This dissociation suggests that mechanisms operating beyond transcriptional regulation, including changes in enzyme activity, membrane localization, substrate availability, or other components of extracellular nucleotide metabolism, may contribute to the observed response. These possibilities remain to be investigated.

Differences between SK-MEL-28 and A375 cells may also reflect their distinct molecular phenotypes. Previous studies have reported substantial differences between melanoma cell lines in ectonucleotidase expression according to tumor stage and cellular origin [69]. In addition, SK-MEL-28 and A375 cells have been characterized as occupying distinct phenotypic states, with SK-MEL-28 cells displaying features associated with a more proliferative, MITF-enriched phenotype, whereas A375 cells exhibit an invasive, MITF-low/AXL-high phenotype and increased expression of mediators associated with tumor progression, including VEGF, MMP-2, and TNF-α [70]. Such differences provide a plausible biological context for the distinct transcriptional and extracellular nucleotide responses observed following melatonin exposure.

The relationship between melatonergic and purinergic signaling may also be considered within the broader concept of the immune-pineal axis, in which inflammatory and purinergic signals interact with melatonergic pathways during cellular and tissue stress [44, 58, 71]. Although the molecular components of this axis were not directly investigated in the present study, the simultaneous occurrence of mitochondrial, inflammatory, and extracellular nucleotide responses raises the possibility of an interaction between melatonergic and purinergic pathways in melanoma cells. Importantly, this interpretation should remain conceptual because the present experimental system did not directly evaluate immune-pineal signaling, NF-κB activation, melatonin synthesis, or receptor-mediated melatonergic responses.

The present findings can also be considered in the context of previous studies linking ectonucleotidase regulation to melanoma-associated inflammatory responses. Rosmarinic acid, for example, has been reported to reduce CD73 expression while increasing inflammatory cytokine production and promoting melanoma cell death [27]. Although the mechanisms of action of rosmarinic acid and melatonin are distinct, both observations support the broader concept that selective alterations in extracellular nucleotide metabolism may accompany changes in inflammatory and tumor-cell responses. This possibility is further supported by clinical evidence associating increased CD73 expression with greater invasiveness and poorer prognosis in metastatic melanoma [72]. Nevertheless, the present study does not establish CD39 or CD73 as determinants of melatonin sensitivity or as prognostic biomarkers, and such applications will require validation in larger experimental and clinical models.

Taken together, the present findings support a concentration- and cell-context-dependent effect of pharmacological melatonin in melanoma cells. Melatonin induced a consistent reduction in metabolic activity and mitochondrial depolarization in both melanoma cell lines, whereas apoptosis-associated, inflammatory, and ectonucleotidase-expression responses differed between SK-MEL-28 and A375 cells. These findings are consistent with the broader concept that melatonin can exert context-dependent cytotoxic or cytoprotective effects in cancer models depending on concentration, exposure conditions, cellular phenotype, receptor expression, and metabolic state [73, 74]. The present study extends this concept by identifying extracellular nucleotide metabolism as an additional cellular response associated with pharmacological melatonin exposure in melanoma cells. Although the present data do not establish a causal link between ectonucleotidase regulation, purinergic receptor signaling, and the observed mitochondrial or inflammatory responses, they provide a basis for further investigation of melatonergic–purinergic interactions in melanoma.

Several limitations should be acknowledged. First, extracellular ATP, ADP, AMP, and adenosine concentrations were not directly quantified; therefore, the consequences of altered nucleotide hydrolysis for extracellular purine availability and adenosine generation remain unresolved. Second, CD39 and CD73 were assessed at the transcriptional and/or enzymatic levels, but protein abundance and membrane localization were not evaluated. Third, purinergic receptor activation, including P2X7 signaling, was not directly investigated. Fourth, changes in apoptosis- and inflammation-associated transcripts were not confirmed at the protein or functional level, precluding definitive conclusions regarding apoptosis, cytokine production, or inflammatory pathway activation. Finally, basal ectonucleotidase expression and activity were not comprehensively characterized before treatment, limiting the extent to which intrinsic differences between SK-MEL-28 and A375 cells can be related to their differential responses. Future studies combining extracellular nucleotide profiling, ectonucleotidase protein and activity measurements, purinergic receptor analysis, and functional assays of cell death and inflammatory signaling will be important to clarify these mechanisms.

Despite these limitations, the present study provides evidence that the cellular response of melanoma cells to pharmacological melatonin extends beyond mitochondrial metabolism and apoptosis-associated gene expression. The findings identify cell-line-dependent changes in extracellular nucleotide metabolism and in CD39/CD73 gene expression as an additional component of the response to melatonin. Importantly, similar mitochondrial alterations were accompanied by distinct transcriptional and extracellular nucleotide responses in SK-MEL-28 and A375 cells, highlighting the influence of cellular context on the biological effects of pharmacological melatonin. Overall, these findings support a model in which melatonin-induced cellular responses in melanoma involve interconnected mitochondrial, inflammatory, and extracellular purinergic processes, while the causal relationships among these pathways remain to be established.

## Conclusion

In conclusion, pharmacological melatonin reduced the metabolic activity of melanoma cells in association with mitochondrial dysfunction, cell-line-dependent transcriptional responses, and alterations in extracellular nucleotide metabolism. These effects were accompanied by differential regulation of CD39/CD73 gene expression, particularly in SK-MEL-28 cells, indicating that pharmacological melatonin can alter extracellular nucleotide processing in melanoma cells and may thereby influence purinergic signaling. To our knowledge, this is the first study to report an association between pharmacological melatonin exposure and changes in CD39/CD73 gene expression in human melanoma cell lines. These findings identify extracellular nucleotide metabolism as a potentially relevant component of the cellular response to pharmacological melatonin and provide a rationale for future studies investigating the mechanistic relationship between melatonergic and purinergic signaling in melanoma.

## Data Availability

No datasets were generated or analysed during the current study.
